# Advertising a Contribution Scheme

**Published:** 1922-06

**Authors:** S. R. Lamb

**Affiliations:** Secretary, Sheffield Joint Hospitals' Council


### Advertising a Contribution Scheme.

*To the Editor of* The Hospital and Health Review.

Sir,—In view of the generous encouragement which you are ever giving to organised schemes for the reconstruction of the voluntary hospitals, I beg to enclose a photograph of two posters which, for the last month, have been displayed by courtesy of the clergy and wardens of St. Paul's Church, immediately facing one of the busiest thoroughfares in this city. The poster was designed by Mr. A. A. Black, of Messrs. Arthur A. Black, Ltd. (hon. director of our Publicity Service). The space and the posting were provided without cost for the hospitals, and the entire amount paid was £6 for the artist's time and material. Over 100,000 contributors have been enrolled, and during the four months ending April 25 this year, £14,500 has been received in contributions under the scheme, being an increase of £10,000 over any preceding year.

The members of this Council sincerely appreciate the part that your journal is taking in the somewhat difficult and delicate task of reconstructing the voluntary hospitals of the country to continue their post-war activities.

Faithfully yours,

S. R. Lamb,

Secretary,

Sheffield Joint Hospitals' Council.

[We regret that the photograph does not lend itself to reproduction on a small scale. The posters are effectively displayed in two lines of bold lettering, side by side over the whole length of the churchyard wall. The inscriptions are, "A penny in the £ from your weekly earnings will secure for you and your dependents free treatment at any of the five Sheffield Hospitals," and "Accidents and illness will happen; but free Hospitals treatment is available only to contributors and their dependents under the Penny in the £ scheme."—Ed.]

